# Abnormal Localization and Tumor Suppressor Function of Epithelial Tissue-Specific Transcription Factor ESE3 in Esophageal Squamous Cell Carcinoma

**DOI:** 10.1371/journal.pone.0126319

**Published:** 2015-05-07

**Authors:** Li Wang, Jie Xing, Rui Cheng, Ying Shao, Peng Li, Shengtao Zhu, Shutian Zhang

**Affiliations:** Department of Gastroenterology & Hepatology, Beijing Digestive Disease Center, Beijing Friendship Hospital, Capital Medical University, National Clinical Research Center for Digestive Diseases, Beijing Key Laboratory for Precancerous Lesion of Digestive Diseases, Beijing, China; Shiga University of Medical science, JAPAN

## Abstract

Esophageal cancer is one of the most common malignant cancers worldwide. The molecular mechanism of esophageal squamous cell carcinoma (ESCC) is still poorly understood. ESE3 is a member of the Ets transcription family, which is only expressed in epithelial tissues and acts as a tumor suppressor gene in prostate cancer. Our study aim was to confirm whether ESE3 is involved in the carcinogenesis of ESCC. Immunohistochemical analysis revealed that ESE3 was mainly located in cell nuclei of normal tissues and the cytoplasm in ESCC tissues. Immunofluorescence and western blot analyses of the normal esophageal cell line HEEpiC and ESCC cell lines EC9706 TE-1, KYSE150, and KYSE410 confirmed these results. pEGFP-ESE3 and pcDNA3.1-V5/HisA-ESE3 plasmids were constructed for overexpression of ESE3 in EC9706 and KYSE150 cells. The stably transfected cells showed restoration of the nuclear localization of ESE3. EC9706 cells with re-localization of ESE3 to the nucleus showed inhibition of proliferation, colony formation, migration, and invasion. To explore the possible mechanism of the differences in localization of ESE3 in normal esophageal cells and ESCC cells, ESCC cell lines were treated with the nuclear export inhibitor leptomycin B, transcription inhibitor actinomycin D, PKC inhibitor sphinganine, P38 MAPK inhibitor SB202190, and CK II inhibitor TBCA. These reagents were chosen according to the well-known mechanisms of protein translocation. However, the localization of ESE3 was unchanged after these treatments. The sequence of ESE3 cDNA in ESCC cells was identical to the standard sequence of ESE3 in the NCBI Genebank database, indicating that there was no mutation in the coding region of ESE3 in ESCC. Taken together, our study suggests that ESE3 plays an important role in the carcinogenesis of ESCC through changes in subcellular localization and may act as a tumor suppressor gene in ESCC, although the mechanisms require further study.

## Introduction

Esophageal cancer is one of the most common malignant cancers in the world. The incidence of esophageal cancer in East Asia including China is much higher than in Western countries with the major pathological type being esophageal squamous cell cancer (ESCC) [1,2]. Early diagnosis and treatment of ESCC are still difficult. The development of ESCC is a very complex process involving multiple genes [3]. Although many genes have been shown to be important in this process, the exact mechanism is poorly understood.

ESE3 is a member of the Ets transcription family and expressed specifically in the nuclei of epithelial cells [4–6]. Studies of prostate cancer indicate that ESE3 is downregulated through promoter methylation and acts as a tumor suppressor gene [7–9]. Previous study has discovered that ESE3 is expressed in the nuclei of normal esophageal epithelial cells [10], however whether ESE3 is involved in the carcinogenesis of ESCC is unknown.

In our study, we found that ESE3 is mainly localized in the cytoplasm of ESCC cells, which differs from its nuclear localization in normal epithelial esophageal cells. We identified ESE3 as a tumor suppressor gene in ESCC and also explored the underlying mechanism for the abnormal localization of ESE3 in ESCC.

## Material and Methods

### Cell culture

ESCC cell lines TE-1 (RIKEN Bio Resource Center, Tsukuba, Japan), EC9706, KYSE150, and KYSE410 (Cancer Institute and Hospital, Chinese Academy of Medical Sciences, China) were cultured in RPMI 1640 medium (Life Technologies, Carlsbad, CA) supplemented with 10% fetal bovine serum (Life Technologies), 100 U/mL penicillin, and 100 mg/mL streptomycin (Life Technologies). The human normal esophageal cell line HEEpiC (ScienCell, San Diego, CA) was cultured in Epicim-2 (ScienCell). All cells were cultured at 37°C with 95% humidity and 5% CO_2_.

### Immunohistochemistry and assessment

ESE3 expression pattern was examined using a tissue microarray (TMA) containing 30 pairs of ESCC tissues and adjacent non-tumor tissues (Outdo Biotech, Shanghai, China) by immunohistochemistry. After deparaffinization in xylene and rehydration through graded ethanol solutions, the TMA was subjected to antigen retrieval by microwave oven heating in 10 mM sodium citrate buffer (pH 6.0) for 15 min. Endogenous peroxidases were inactivated by incubation in 3% hydrogen peroxide for 15 min and nonspecific binding sites were blocking in 10% normal goat serum for 15 min. Then, a rat monoclonal anti-ESE3 antibody (1:100; Lifespan, USA) was applied as the primary antibody at 4°C overnight, followed by incubation with a biotin-conjugated secondary antibody for 30 min and then streptavidin peroxidase for 15 min. The TMA was washed in PBS three times for 5 min each wash between incubation steps, and color was developed using diaminobenzidine substrate. After counterstaining with hematoxylin and dehydration in 100% ethanol and xylene, the TMA was mounted with glycerol-gelatin. The expression pattern of ESE3 was then examined by light microscopy.

The positivity rate of ESE3 in each sample was calculated by randomly observing at least three high power fields with 100 epithelial cells in each. Subcellular localization of ESE3 was determined as follows: nuclear, defined as positive signals mainly in the nucleus; cytoplasmic, defined as positive signals mainly in the cytoplasm; nuclear-cytoplasmic, defined as evenly distributed positive signals.

### Immunofluorescence and live cell imaging

ESCC cell lines and HEEpiC cells were grown on glass coverslips (NEST Biotechnology, China) to 70% confluence and then fixed in 4% paraformaldehyde for 15 min at room temperature. The cells were permeabilized with 0.3% Triton X-100 for 20 min, blocked with 5% bovine serum albumin for 30 min, and then incubated with the rat monoclonal anti-ESE3 antibody at 4°C overnight, followed by an Alexa Fluor 488-conjugated secondary antibody (Jackson, USA) for 45 min at 37°C. The cells were rinsed in PBS between incubation steps and mounted with medium containing 4'-6-diamidino-2-phenylindole. Both fixed and living cells transfected with an enhanced green fluorescent protein (EGFP) expression plasmid were examined under an IX51 fluorescence microscope (Olympus, Japan).

### Western blot analysis

NE-PER Nuclear and Cytoplasmic Extraction Reagents (Thermo Pierce, USA) were employed according to the manufacturer’s instructions to prepare nuclear and cytoplasmic protein extracts. Lamin-B1 and α-tubulin were used as reference proteins for nuclear and cytoplasmic proteins, respectively. Equal amounts of protein (20–40 μg/lane) were subjected to 10% SDS polyacrylamide electrophoresis. Separated proteins were then transferred to polyvinylidene fluoride membranes (Millipore, USA). After incubation with TBST containing 5% non-fat dry milk for 2 h at room temperature, the blots were incubated with the indicated primary antibodies at 4°C overnight, followed by washing in TBST and incubation with a horseradish peroxidase-conjugated secondary antibody for 1 h at room temperature. Protein bands were developed using Immobilon Western Chemiluminescent HRP Substrate (Millipore). The following antibodies were used in the experiments: rat monoclonal anti-ESE3 antibody, rabbit monoclonal anti-α-tubulin antibody, and rabbit monoclonal anti-lamin-B1 antibody (Cell Signaling Technology, USA).

### Cell proliferation analysis

Cell proliferation was determined by the 3-(4,5-methylthiazol-2-yl)-2,5-diphenyltetrazolium bromide (MTT) method. Briefly, ESE3- and empty vector-transfected EC9706 cells as well as untransfected EC9706 cells were seeded in 96-well plates at a density of 1000 cells per well and incubated at 37°C with 5% CO_2_. At 24, 48, 72, 96, and 120 h, the medium was removed and the cells were incubated with serum-free RPMI 1640 medium containing 0.2 mg/mL MTT for 4 h. Then, the supernatant was discarded and 150 μL dimethyl sulfoxide was added per well. The plates were vortexed lightly for 10 min to solubilize the MTT formazan completely and then the optical density of each well was measured on a microplate reader (Smartspec Model 450; Bio-Rad Laboratories Inc., USA) at 490 nm. Each experiment was repeated at least three times and in six wells for each experiment.

### Cell apoptosis analysis

Early apoptosis rates of cells were measured using an Annexin V-PE/7-AAD Kit (Becton Dickinson, USA) following the manufacturer’s instructions. Briefly, cells were harvested, washed with cold PBS, and resuspended in binding buffer. Then, 5 μL Annexin V-PE and 5 μL 7-AAD were added to each sample containing 1×10^5^ cells/100 μL. The samples were incubated at 25°C in the dark for 15 min, followed by addition of 400 μL binding buffer. Within 1 h of preparation, the samples were analyzed by a flow cytometer (Attune; Applied Biosystems). The experiments were repeated three times independently.

### Cell-cycle analysis

Cell-cycle analysis was performed through measuring DNA content by flow cytometry using propidium iodide (PI) (Sigma aldrich, USA). Briefly, cells were harvested, fixed in 75% cold ethanol, and stored at 4°C for at least overnight. Then, the cells were washed in PBS and treated with 100 μg/mL ribonuclease A (Roche) and 50 μg/mL PI at 37°C for 1 h. The percentages of cells were compared in G0/G1, S, and G2/M phases. The experiments were repeated three times.

### Colony formation assay

An anchorage-dependent colony formation assay was performed to assess the survival and proliferation of ESCC cells. Briefly, cells were trypsinized and resuspended as single cells in RPMI 1640 medium. The cells were seeded at a density of 200 cells/10 mL in a 6×6 cm plate and incubated at 37°C in a humidified atmosphere with 5% CO_2_ for 14–27 days or until the appearance of colonies. Finally, the cell colonies were fixed with methanol, stained with Giemsa (Sigma-Aldrich), and then counted under a microscope. The experiment was performed in triplicate plates and each experiment was repeated three times.

### In vitro migration and invasion assays

In vitro migration assays were performed using a 6.4-mm BD chamber (Becton Dickinson) containing a polyethylene terephthalate membrane filter with an 8-μm pore size. In vitro invasion assays were performed using a 6.4-mm BD BioCoat^.^ Matrigel Invasion Chamber (Becton Dickinson) containing a matrigel-precoated, polyethylene terephthalate membrane filter with an 8-μm pore size. Briefly, 5×10^4^ EC9706 cells were resuspended in 0.5 mL serum-free RPMI 1640 medium and then seeded on the upper chamber. Then, 0.75 mL complete RPMI 1640 medium was added to the lower chamber as the chemoattractant. After incubation for 36–48 h at 37°C in a humidified atmosphere with 5% CO_2_, non-migratory or non-invasive cells on the upper surface of the membrane were removed mechanically. Cells on the bottom surface were then fixed with methanol, stained with hematoxylin and eosin, and counted as migratory or invasive cells under a microscope. The assays were repeated three times.

### RNA isolation, cDNA synthesis, plasmid construction, and establishment of stably transfected clones

Total RNA was extracted from cells using Trizol (Invitrogen, USA). cDNA synthesis from 2 μg total RNA was performed using the SuperScript III Reverse Transcriptase System (Invitrogen) and an oligo(dT)18 Primer in a 20-μL reaction volume. The ESE3 coding sequence was amplified using the primers listed in (S1 Table) and then subcloned into pcDNA3.1-V5/HisA, pEGFP-N1, and pEGFP-C3 expression vectors. Plasmids were transfected into EC9706 and KYSE150 cells using Lipofectamine 2000 (Invitrogen) according to the manufacturer’s instructions. Briefly, cells at 90% confluence were washed with serum-free medium and then treated with DNA-Lipofectamine 2000 complexes consisting of 4 μg ESE3 plasmid and 10 μL Lipofectamine 2000. After incubation for 5 h, the complexes were washed out and complete medium with serum was added to the cells. At confluency, the cells were subcultured at a split ratio of 1:10 into selection medium (complete medium plus G418) for stable transfectant selection. Empty expression vectors (pcDNA3.1/V5-His A and pEGFP) were also transfected into cells as controls. Stably transfected cells were routinely cultured in selection medium.

### Statistical analysis

All statistical analyses were performed using SPSS software 13.0 (SPSS Inc., Chicago, IL, USA). Differences between groups were assessed by the t-test or chi-square test. A value of P<0.05 was considered statistically significant. All data represent at least three independent experiments. Results are expressed as the mean ± standard deviation (SD).

## Results

### Subcellular localization of ESE3 differs in normal esophageal epithelial tissues and ESCC tissues

**Fig 1:**
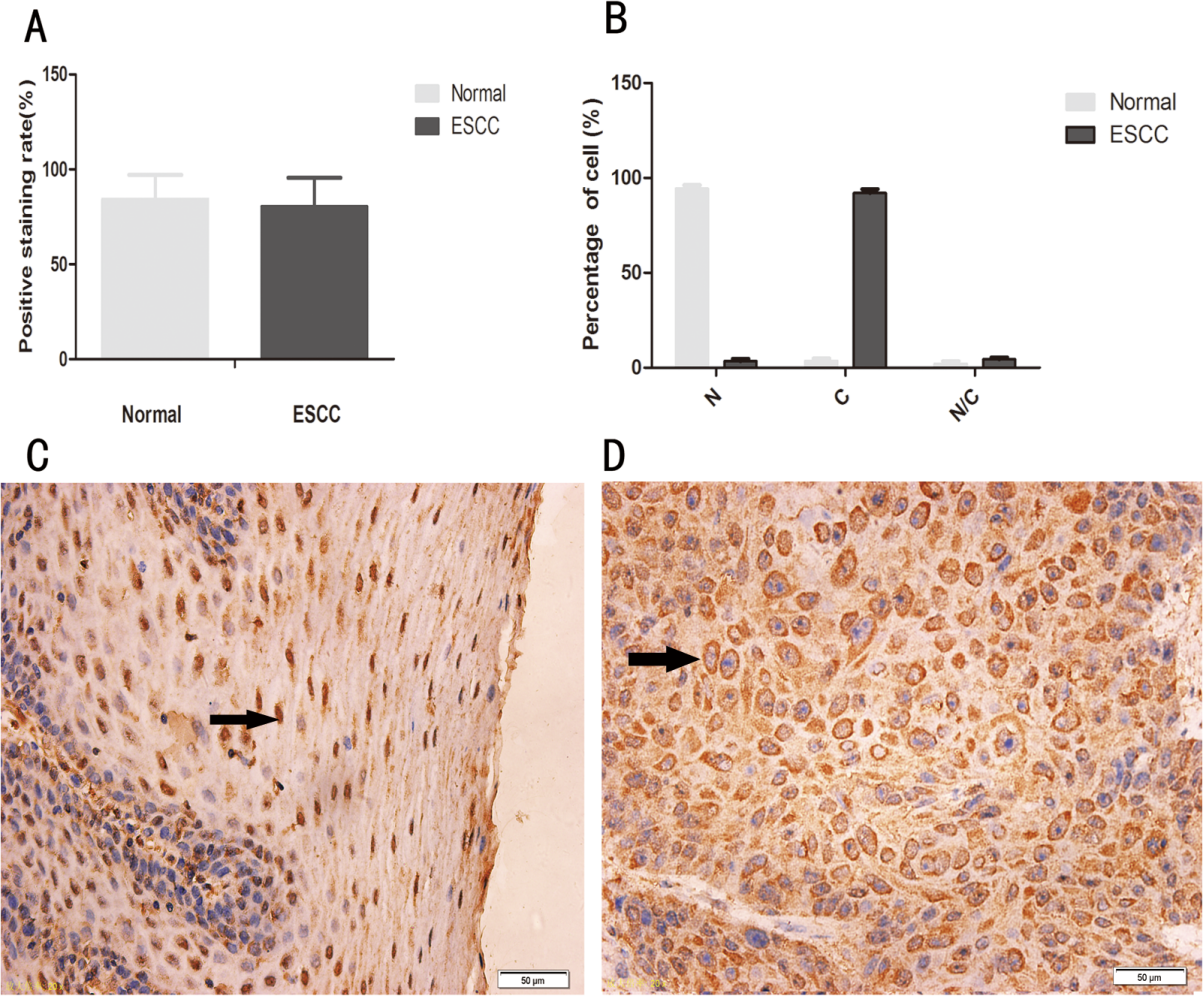
Expression and subcellular localization of ESE3 in normal human esophageal epithelial tissues and ESCC tissues. (A) Positivity rate in normal human esophageal epithelial tissues and ESCC tissues (P>0.05). (B) Subcellular localization of ESE3 in normal esophageal epithelial cells and ESCC cells. N indicates nuclear localization, C indicates cytoplasmic localization, and N/C indicates nuclear/cytoplasmic localization (equal distribution or uncertain localization). The white bar represents normal esophageal epithelial cells, and the black bar represents ESCC cells (P<0.05). (C) Positive staining of ESE3 in the nucleus of normal esophageal epithelial cells. (D) Positive staining of ESE3 in the cytoplasm of ESCC cells.

We first examined the expression and subcellular localization of ESE3 in a TMA containing 30 pairs of ESCC tissues and adjacent normal esophageal epithelial tissues by immunohistochemical staining. Positive staining for ESE3 was observed in all samples including normal esophageal epithelial tissues (30/30) and ESCC tissues (30/30) with comparable positivity rates (84.4% ± 12.7% vs 80.6% ± 14.9%, P>0.05), whereas ESE3 exhibited distinct subcellular localization patterns in normal esophageal epithelial cells compared with those in ESCC cells. ESE3 was mainly localized in the nuclei of normal esophageal epithelial cells and in the cytoplasm of ESCC cells (Fig 1).

**Fig 2:**
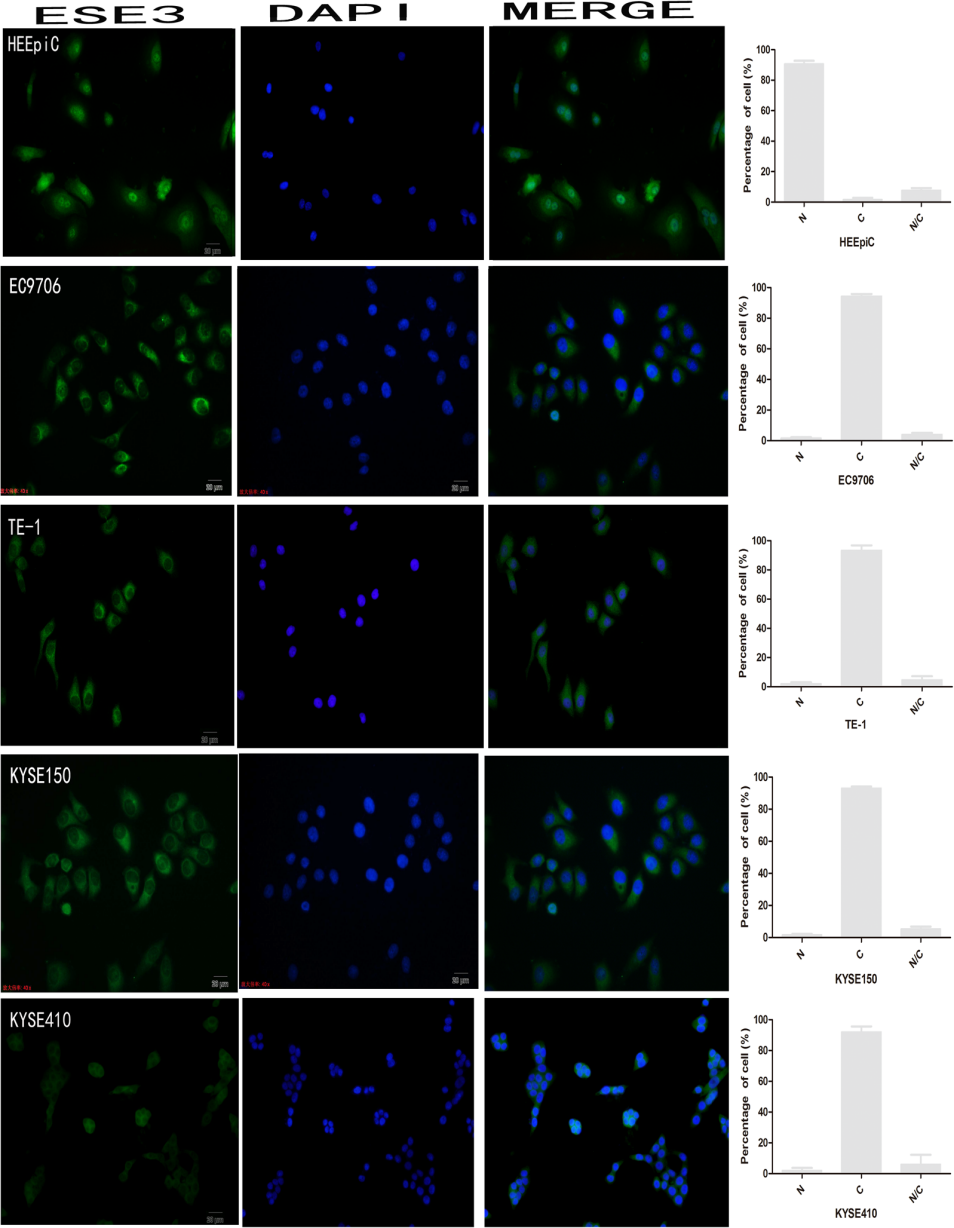
Subcellular localization of ESE3 in esophageal cell lines by immunofluorescence analysis. N indicates nuclear localization, C indicates cytoplasmic localization, and N/C indicates nuclear/cytoplasmic localization (equal distribution or uncertain localization).

**Fig 3:**
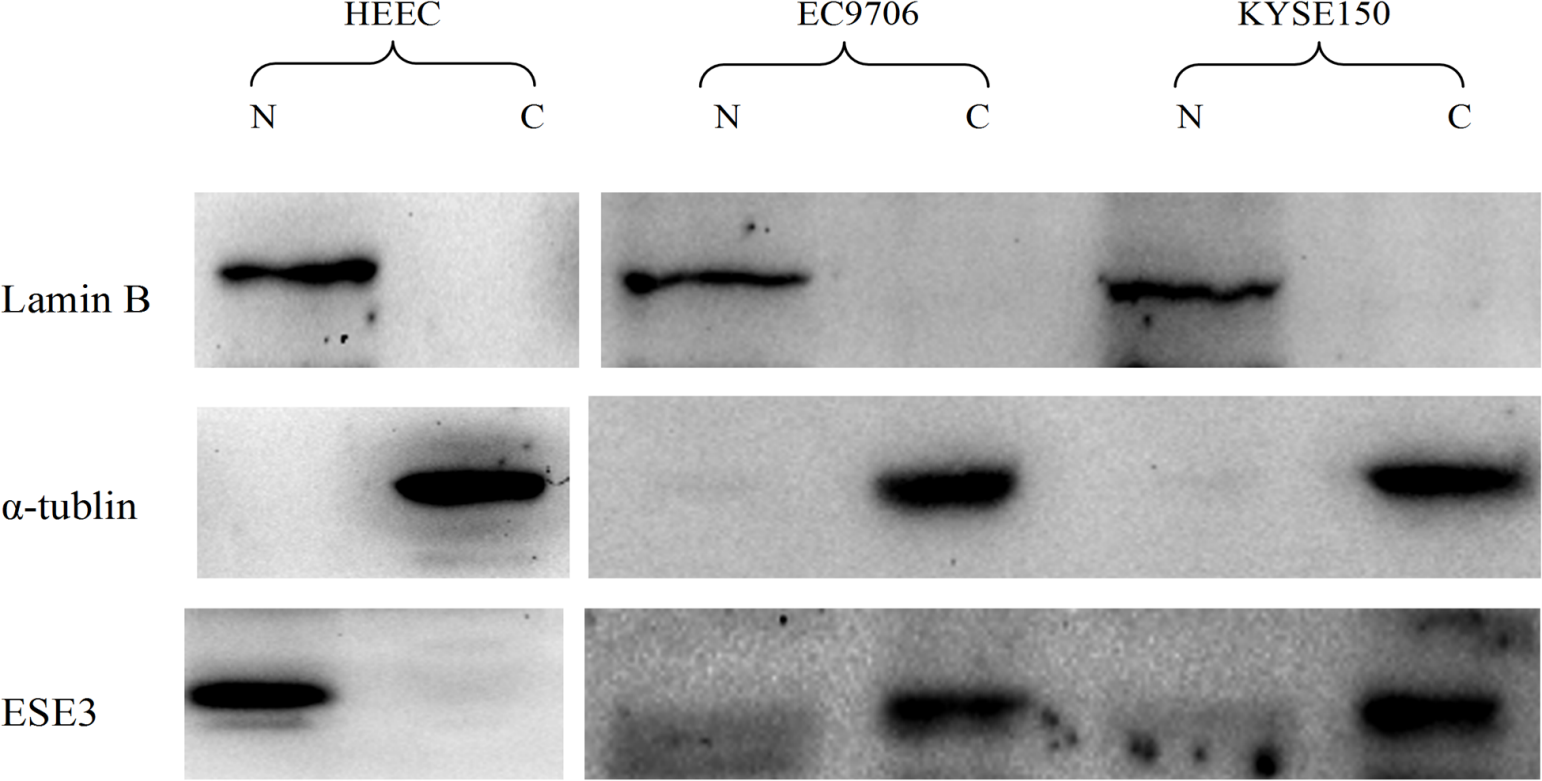
Western blot analysis of ESE3 localization. Nuclear and cytoplasmic protein extracts were verified by detection of lamin B1 and α-tubulin, respectively. ESE3 was found in the nuclear protein extracts of HEEpiC cells and in the cytoplasmic protein extracts of EC9706 cells.

The localization of ESE3 in normal esophageal epithelial cells and ESCC cells was also examined by immunofluorescence. The results showed that ESE3 was expressed in all cell lines and localized mainly in the nuclei of HEEpiC cells and the cytoplasm of ESCC cell lines (Fig 2). Nuclear and cytoplasmic proteins of the normal esophageal epithelial cell line HECC and esophageal cell line EC9706 were extracted and analyzed by western blotting to confirm the subcellular localization of ESE3. The results were consistent with the findings of immunohistochemical and immunofluorescence analyses (Fig 3).

### Overexpression of ESE3 leads to nuclear re-localization in ESCC

**Fig 4:**
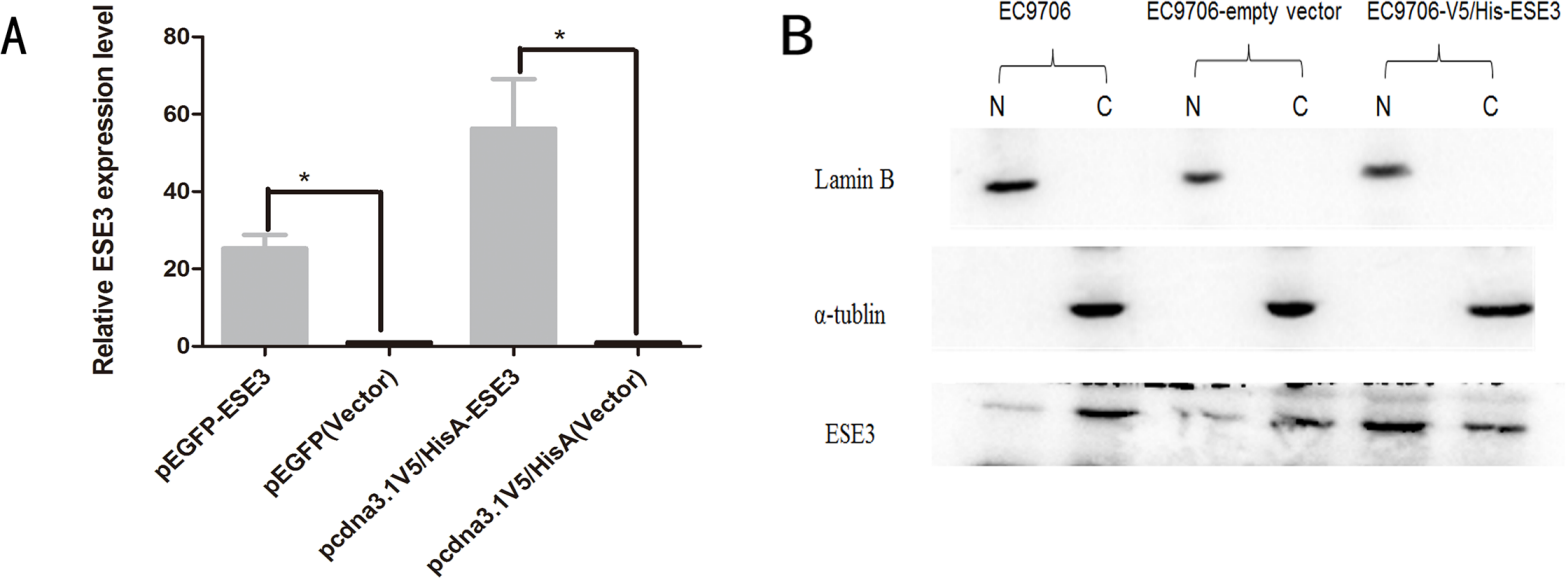
Localization of overexpressed ESE3. **(A) RT-PCR analysis showed that ESE3 was overexpressed in pEGFP-ESE3- and pcdna3.1-V5/HisA-ESE3-transfected EC9706 cells**. (B) Localization of ESE3 in pcdna3.1-V5/HisA-ESE3-transfected EC9706 cells by western blot analysis.

**Fig 5:**
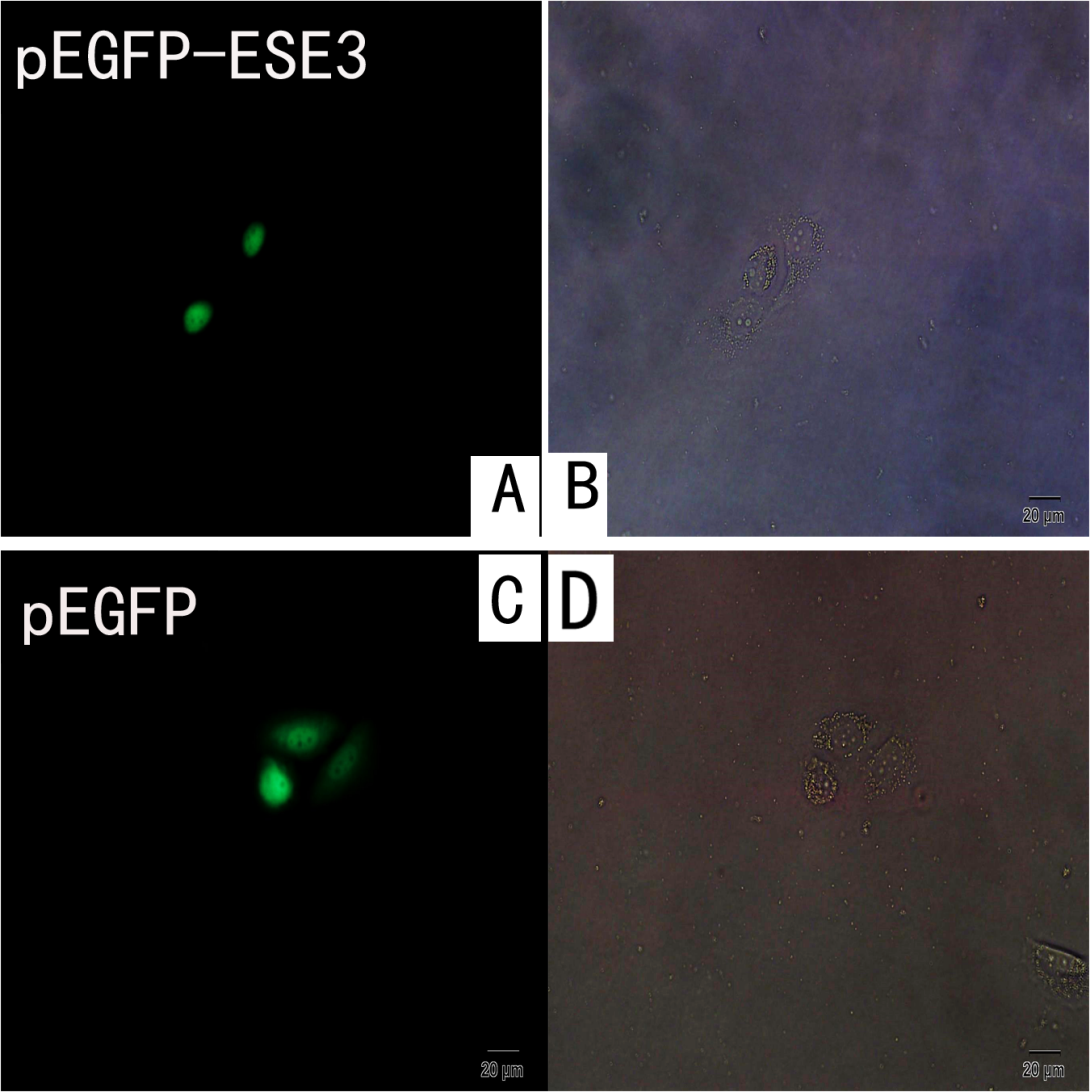
Localization of overexpressed ESE3 in living pEGFP-ESE3-transfected EC9706 cells. (A) location of overexpressed ESE3-EGFP in living pEGFP-ESE3-transfected EC9706 cells by fluorescence microscope. (B) image of pEGFP-ESE3-transfected EC9706 cells by phase contrast microscope. (C) location of EGFP in living vector-transfected EC9706 cells by fluorescence microscope. (D) image of pEGFP-transfected EC9706 cells by phase contrast microscope.

**Fig 6:**
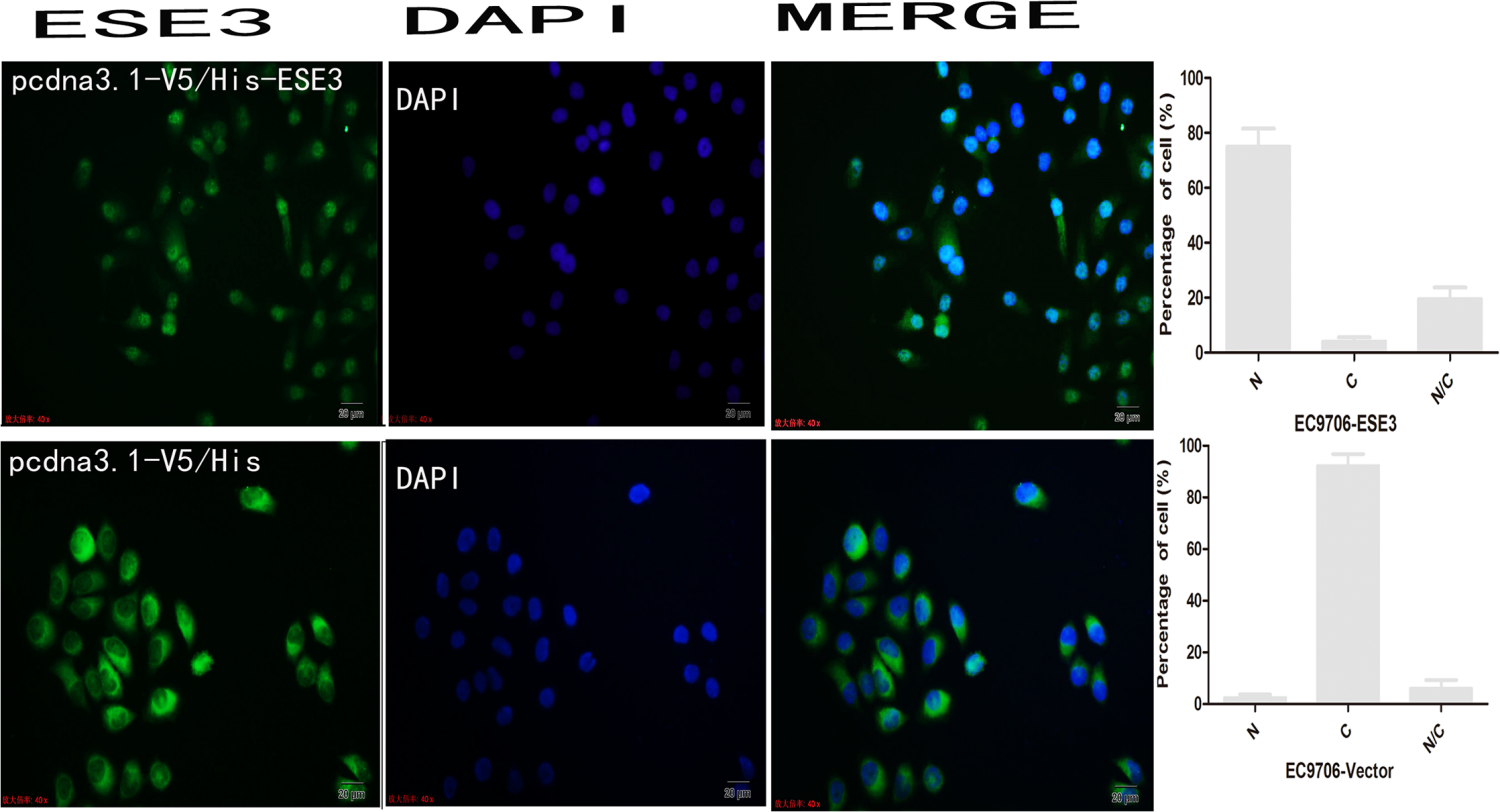
Immunofluorescence analysis of the localization of ESE3 in pcdna3.1-V5/HisA-ESE3-transfected EC9706 cells.

To observe the localization of ESE3, pEGFP-N1/ESE3 and pEGFP-C3/ESE3 were stably transfected into EC9706 and KYSE150 cells. EGFP was fused to either the N- or C-terminus of ESE3 to assess mislocation of the ESE3-EGFP fusion protein. Additionally, to rule out the influence of EGFP on the localization of ESE3, we constructed pcDNA3.1-V5/HisA-ESE3 to express ESE3 with a much smaller tag. All plasmids were sequenced and RT-PCR was carried out to confirm successful transfection of the constructs. The localization of EGFP-tagged ESE3 including ESE3-EGFP-N1 and ESE3-EGFP-C3 in EC9706 and KYSE150 cells was examined by fluorescence microscopy. The localization of V5-tagged ESE3 was examined by immunofluorescence staining. Surprisingly, the overexpressed ESE3 from the three different plasmids was localized in the nuclei of both transiently and stably transfected EC9706 and KYSE150 cells, which was different to the cytoplasmic localization of endogenous ESE3 in ESCC cells (Figs 4, 5 and 6). ESE3a and ESE3b are two isoforms of ESE3 with slight differences in esophageal cells and both of these isoforms were cloned into plasmids. Immunofluorescence analysis showed no differences in the localization of the two isoforms (data not shown).

### Re-localization of ESE3 to the nucleus in ESCC cells inhibits proliferation, colony formation, migration, and invasion

**Fig 7:**
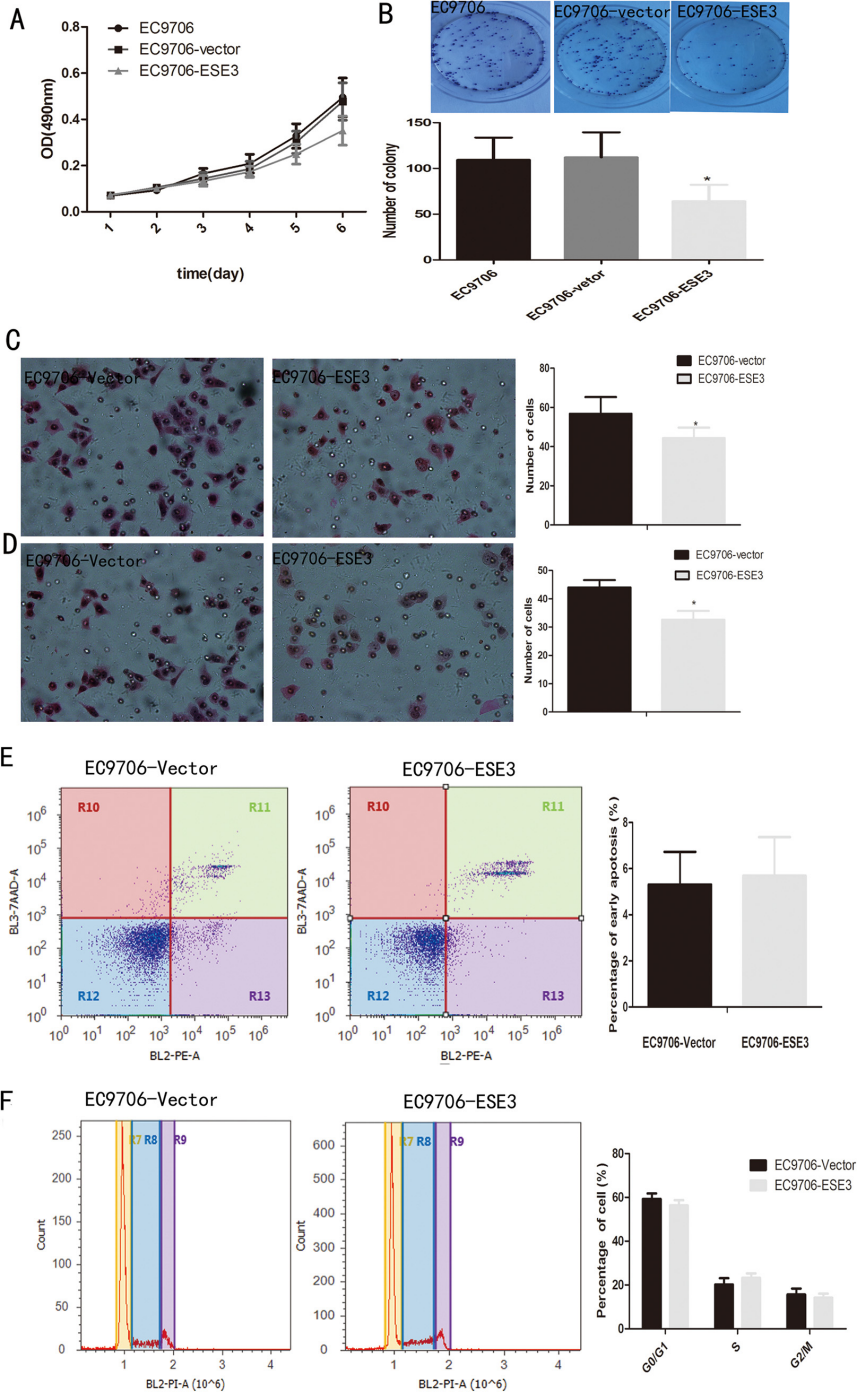
Effects of ESE3 re-localization to the nuclei of EC9706 cells. (A) Cell proliferation was inhibited in EC9706 cells transfected with pcdna3.1-V5/His-ESE3 in MTT assays. (B) Fewer colonies were formed by EC9706 cells transfected with pcdna3.1-V5/His-ESE3 compared with control groups. (C) Migratory or (D) invasive cells on the bottom surface were fixed, stained with hematoxylin and eosin, and counted under a microscope at 200×. Data are the means ± SD of three independent experiments (*P<0.05). (E) and (F) shows no difference in apoptosis or the cell-cycle distribution of the two groups.

Because overexpression of ESE3 restored its nuclear localization in ESCC cells, we further examined the physiological significance of recovery of the subcellular localization of ESE3. First, we examined cell proliferation by MTT assays. EC9706 cells overexpressing ESE3 in the nucleus showed a decrease in cell proliferation compared with empty vector-transfected and untransfected EC9706 cells (Fig 7A), indicating that ESE3 negatively regulates the proliferation of ESCC cells. In addition, EC9706 cells overexpressing ESE3 showed a decrease in colony formation compared with empty vector-transfected and untransfected EC9706 cells (Fig 7B). Cell migration and invasion were measured by two types of modified Boyden chamber. A chamber containing a polyethylene terephthalate membrane filter was used to examine the migration of ESCC cells. Compared with empty vector-transfected EC9706 cells, re-localization of ESE3 to the nucleus resulted in significantly fewer migratory cells (Fig 7C). A modified Boyden chamber containing a polyethylene terephthalate membrane coated with matrigel was used to investigate the invasion of ESCC cells. Similarly, compared with empty vector-transfected EC9706 cells, fewer ESE3-transfected EC9706 cells invaded through the matrigel-coated membrane (Fig 7D).

Furthermore, we investigated the effect of re-localization of ESE3 to the nucleus on the cell cycle and apoptosis. The results showed no significant difference between ESE3-transfected and empty vector-transfected EC9706 cells in cell-cycle or apoptosis assays (Fig 7E and 7F).

### Mechanism of nuclear localization of ESE3 in ESCC cells

**Fig 8:**
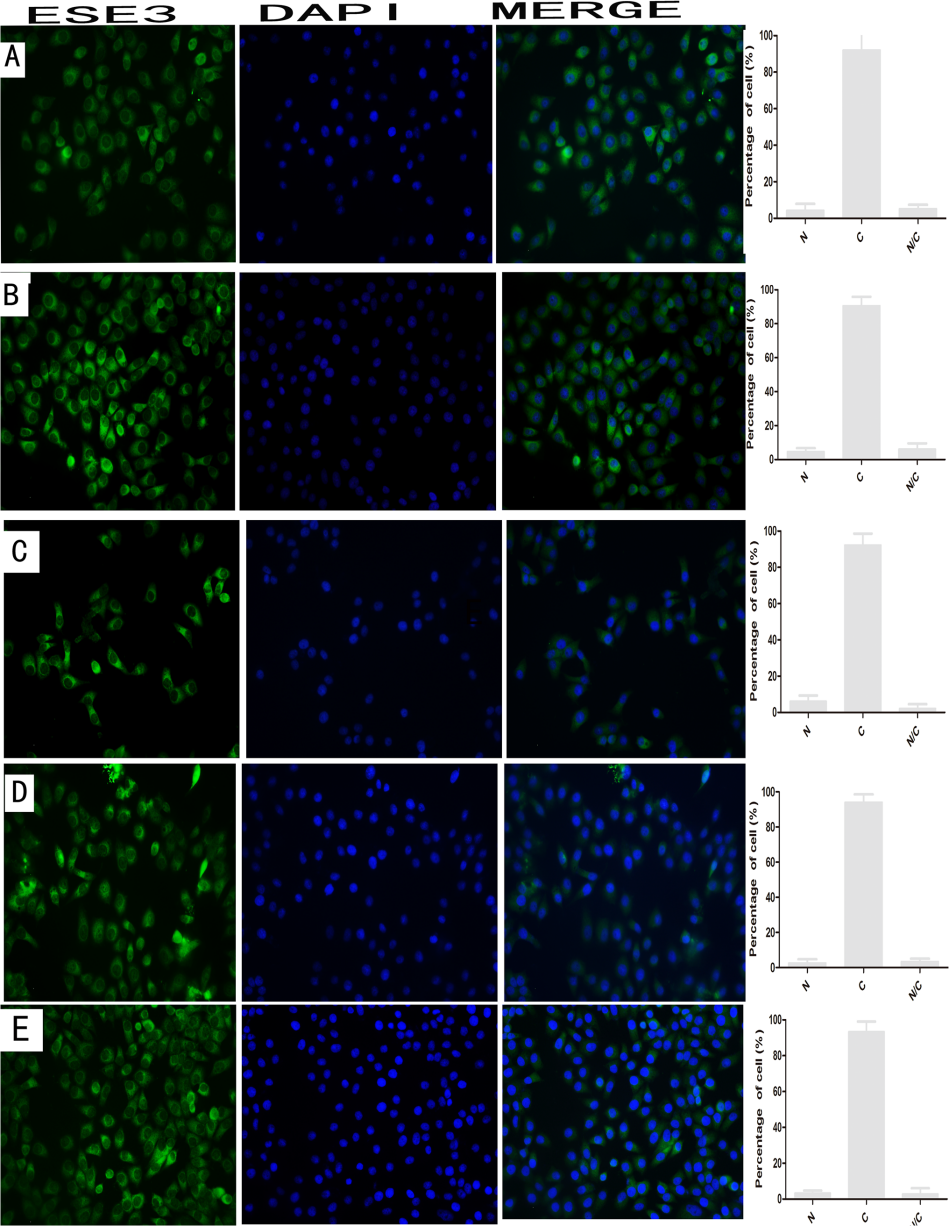
Subcellular localization of ESE3 in EC9706 after treatment by immunofluorescence analysis. (A), (B), (C), (D) and (E) indicate subcellular localization of ESE3 after treatment by leptomycin B, actinomycin D, sphinganine (pKC inhibitor), SB202190 (pk38 MAPK inhibitor) and TBCA (CK II inhibitor), respectively. N indicates nuclear localization, C indicates cytoplasmic localization, and N/C indicates nuclear/cytoplasmic localization (equal distribution or uncertain localization).

To further explore the mechanism of translocation of ESE3 from the nucleus to the cytoplasm, we examined well-known protein relocation pathways. EC9706 cells were first treated with leptomycin B, which inhibits nuclear export mediated by CRM1, and actinomycin D that inhibits transcription-mediated protein nuclear export. ESE3 showed no obvious change in localization after these treatments. To exclude the possibility that phosphorylation of ESE3 changes its location, we treated EC9706 cells with sphinganine (pKC inhibitor), SB202190 (pk38 MAPK inhibitor), and TBCA (CK II inhibitor) according to possible kinase phosphorylation predicted by NetNET1.1. ESE3 showed no obvious change in location after these treatments, indicating that localization of ESE3 is not mediated by the associated kinases (Fig 8).

## Discussion

The Ets gene family plays an important role in multiple cell processes including cell proliferation, differentiation, migration, and apoptosis [5,6]. ESE3 is specifically expressed in epithelial cells and thought to be involved in many diseases [11]. Previous studies have revealed that ESE3 acts as a transcriptional repressor and tumor suppressor gene in prostate epithelial cells. It is downregulated by promoter methylation, leading to inhibition of cell apoptosis in prostate cancer [7]. Moreover, ESE3 controls the expression of NKx3.1 and epithelial-to-mesenchymal transition-related genes such as ZEB2, BMI1, and POU5F1 [8,9]. Although there are few studies of ESE3 at present, ESE3 has exhibited tumor suppressor characteristics. In our study, we investigated whether ESE3 is involved in the carcinogenesis of ESCC.

In TMA analysis, we found that ESE3 was mainly localized in the cytoplasm of ESCC cells, which was different from its nuclear localization in normal esophageal epithelial cells. Immunofluorescence analysis of ESCC cell lines confirmed the results of western blot analysis of nuclear/cytoplasmic protein extracts. Similar to other transcription factors [12,13], the change in subcellular localization of ESE3 probably leads to transcriptional changes in ESCC. Therefore, our findings suggest a new mechanism of transcriptional regulation by ESE3.

To observe the localization of ESE3 in living ESCC cells, we performed plasmid transfection in EC9706 and KYSE150 cells. EGFP-tagged ESE3 showed distinct nuclear localization compared with the even distribution of ESE3 in empty vector-transfected ESCC cells. Both N- and C-terminal tagged EGFP-ESE3 showed the same nuclear localization. Additionally, the localization of ESE3 with the relatively smaller V5 tag in V5-HisA plasmid-transfected ESCC cells was nuclear, indicating that the nuclear localization of overexpressed ESE3 was not caused by the EGFP tag. We also constructed plasmids to express two ESE3 isoforms expressed in esophageal epithelial cells [14]. The localization of the ESE3a isoform was the same nuclear localization as the ESE3b isoform in transfected ESCCs, indicating the different isoforms were not responsible for the difference in the localization of endogenous and exogenous ESE3.

Exogenous and overexpressed ESE3 was re-localized to the nucleus, although the exact mechanism was unclear. We evaluated the effects of ESE3 re-localization to the nuclei of ESCC cells by cell proliferation, colony formation, migration, invasion apoptosis, and cell-cycle analyses. Consistent with the previous findings in prostate cancer, EC9706 cells with re-localization of ESE3 to the nucleus showed impairments in cell proliferation, colony formation, migration, and invasion. However, ESE3 had no obvious effect on the cell cycle or apoptosis as measured by flow cytometric analysis. These results suggest that ESE3 might act as a tumor suppressor gene and play an important role in ESCC. However, the molecular mechanism of ESE3 in these processes requires further elucidation.

We also explored the underlying mechanism of cytoplasmic localization of ESE3 in ESCC. Unfortunately, the localization of ESE3 was unchanged after treatments with leptomycin B, actinomycin D, and some kinase inhibitors, indicating that the molecular mechanism of abnormal localization of ESE3 was mediated through an unknown pathway [15–17]. The sequence of ESE3 cDNA in the ESCC cell lines was the same as that in the NCBI Genebank database, suggesting that the cytoplasmic localization of ESE3 in tumors was not caused by somatic mutation. Possibly, ESE3 is retained in the cytoplasm because of protein interactions [18]. Another possible explanation for the different localizations of endogenous and exogenous ESE3 in ESCC cells is that the cells were overwhelmed by the overexpressed ESE3, resulting in saturation of the nuclear export system and retention of ESE3 in the nucleus. The exact mechanism of abnormal cytoplasmic localization of ESE3 in ESCC requires further study.

In summary, our study revealed translocation of ESE3 into the cytoplasm of ESCC cells, which acts as a tumor suppressor gene in the carcinogenesis of ESCC. The nuclear localization of ESE3 can be restored by its overexpression. This re-localization of ESE3 is possibly caused by an alteration in protein interactions or saturation of the nuclear export system. Restoration of the ESE3 localization inhibits ESCC cell growth and colony formation, suggesting a possible novel mechanism of ESCC carcinogenesis by ESE3 re-localization.

## Supporting Information

S1 Table Primers used in plasmid construction. (DOCX)

S1 Fig Whole slide picture of TMA. (TIF)

S2 Fig Low magnification of cell expression of ESE3. (A) Subcellular localization of ESE3 in normal esophageal cells, (B) Subcellular localization of ESE3 in ESCC cells.
(TIF)
