# Supplementary material for: Abnormal Localization and Tumor Suppressor Function of Epithelial Tissue-Specific Transcription Factor ESE3 in Esophageal Squamous Cell Carcinoma
Source: PLoS One. 2015 May 7;10(5):e0126319. doi: 10.1371/journal.pone.0126319 (PMC4423989; doi:10.1371/journal.pone.0126319)
Supplement: S1 Table — (DOCX) [file pone.0126319.s001.docx]

 Supporting table

Table Primers used in plasmid construction

| ESE3-EGFP-N1 | 5′-CCCAAGCTTATGATTCTGGAAGGAGGTGGTGTAATG-3′ |
| --- | --- |
|  | 5′-GCGGGATCCAGGTTTTCATTTTCTCTCCATCCTCGGGCAT-3′ |
| ESE3-EGFP-C3 | 5′-CCCAAGCTTATGATTCTGGAAGGAGGTGGTGTAAT-3′ |
|  | 5′-GCGGGATCCTCAGTTTTCATTTTCTCTCCATCCT-3′ |
| ESE3-V5-HisA | 5′-CCCAAGCTTGCCACCATGATTCTGGAAGGAGGTGGTGTAATGAA-3′ |
|  | 5′-GCGGGATCCGGTTTTCATTTTCTCTCCATCCTCGGGCAT-3′ |
