# Supplementary figures and images for: Abnormal Localization and Tumor Suppressor Function of Epithelial Tissue-Specific Transcription Factor ESE3 in Esophageal Squamous Cell Carcinoma
Source: PLoS One. 2015 May 7;10(5):e0126319. doi: 10.1371/journal.pone.0126319 (PMC4423989; doi:10.1371/journal.pone.0126319)

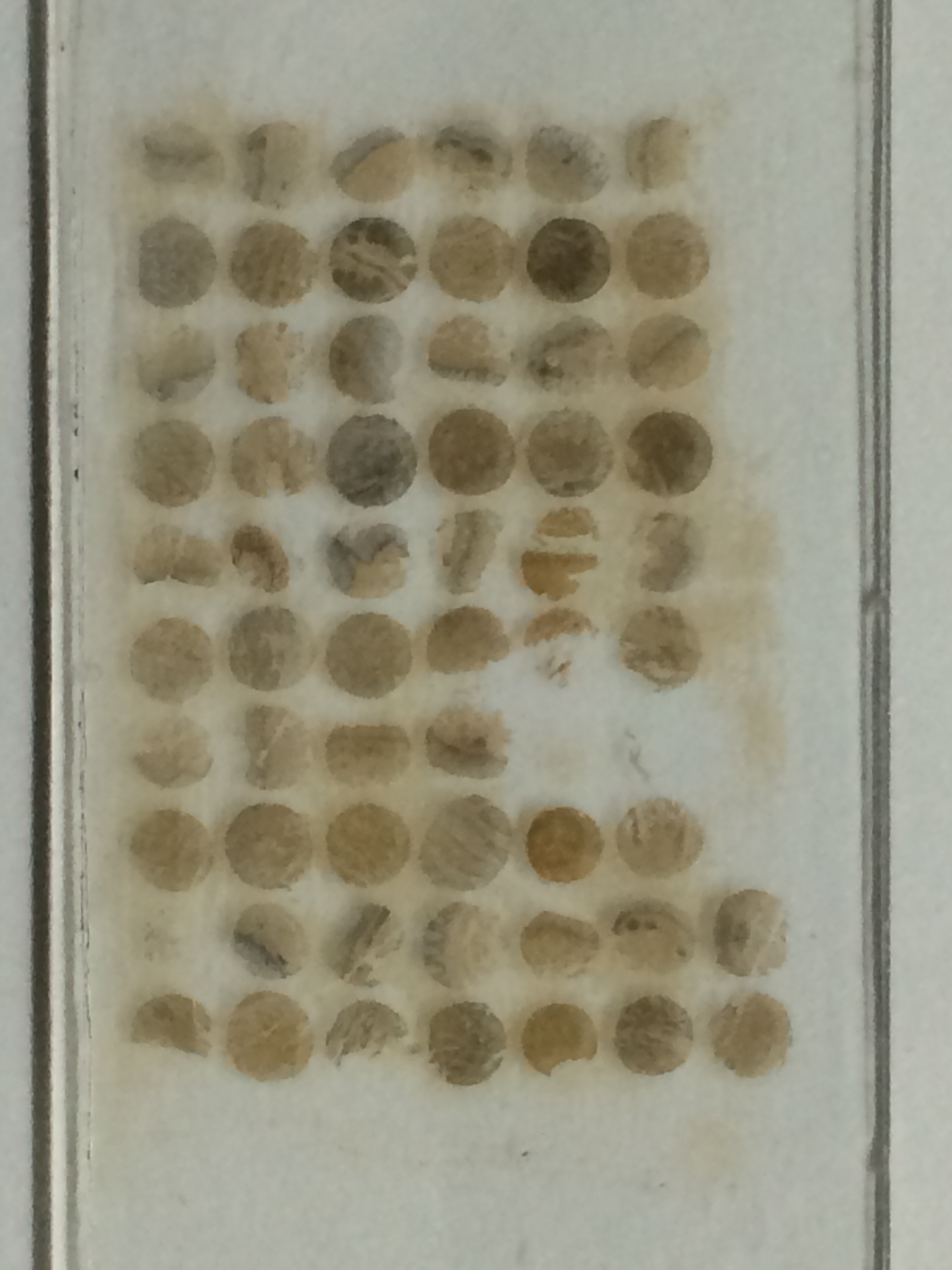

Supplement: S1 Fig — (TIF) [file pone.0126319.s002.tif]

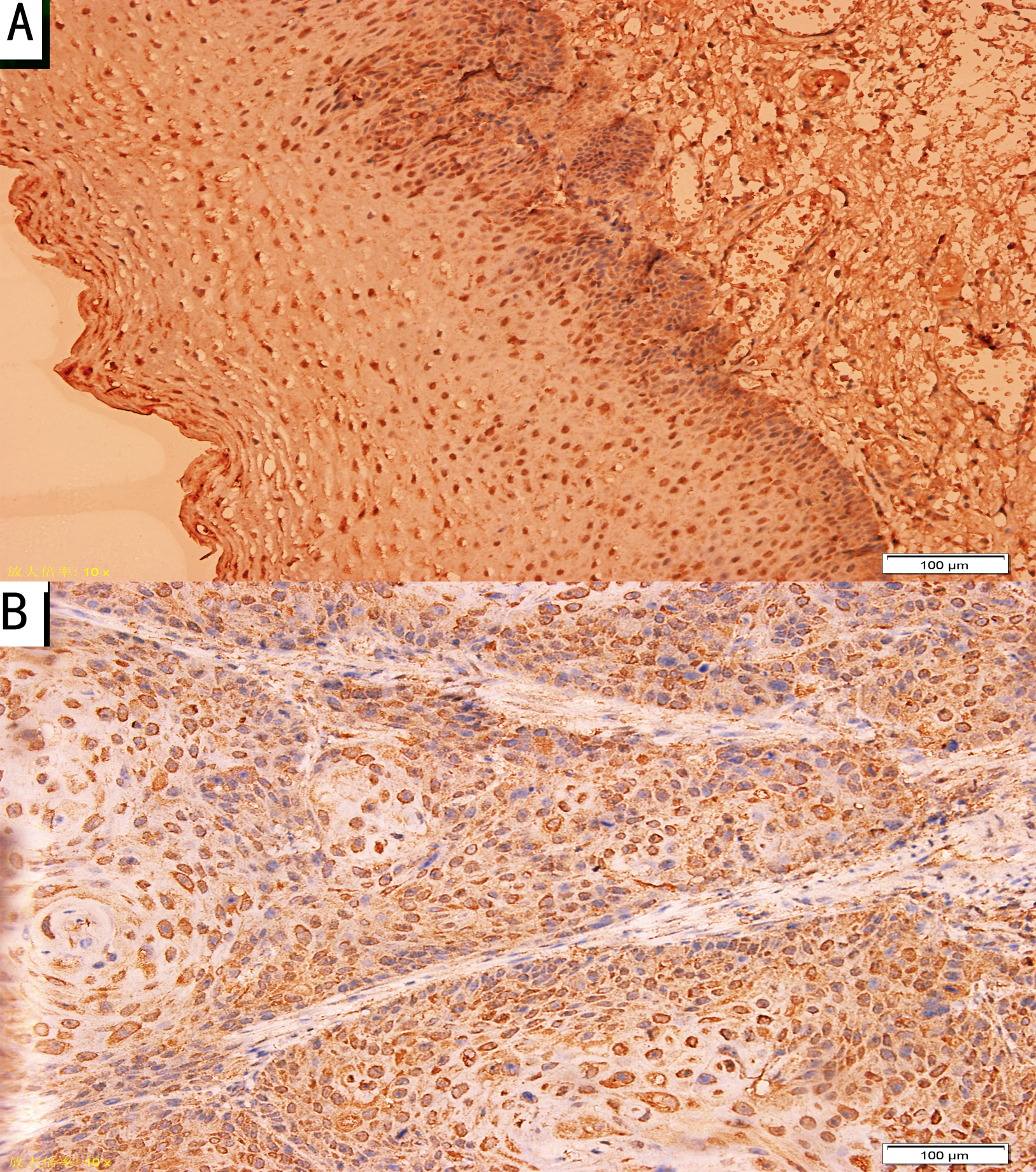

Supplement: S2 Fig — (A) Subcellular localization of ESE3 in normal esophageal cells, (B) Subcellular localization of ESE3 in ESCC cells. (TIF) [file pone.0126319.s003.tif]
